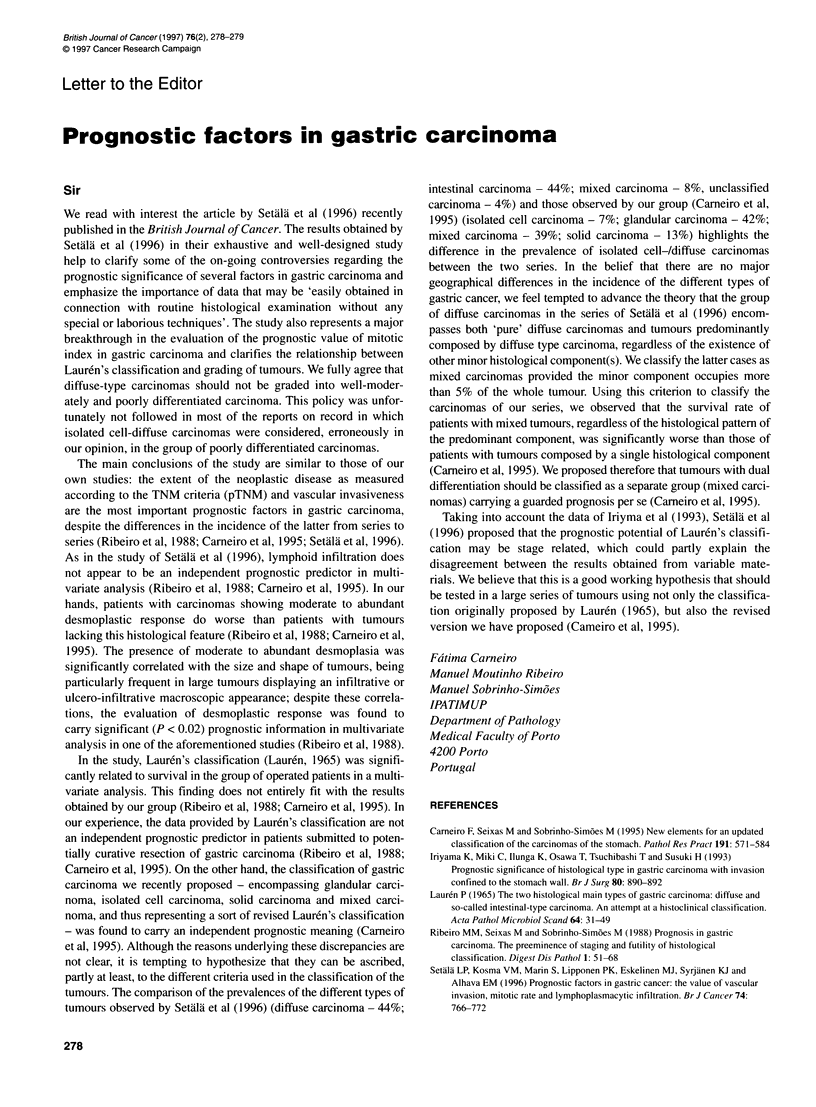# Prognostic factors in gastric carcinoma.

**DOI:** 10.1038/bjc.1997.376

**Published:** 1997

**Authors:** F. Carneiro, M. M. Ribeiro, M. Sobrinho-Simoes


British Journal of Cancer (1997) 76(2), 278–279
© 1997 Cancer Research Campaign

Letter to the Editor

### Prognostic factors in gastric carcinoma

### Sir

We read with interest the article by Setälä et al (1996) recently published in the *British Journal of Cancer*. The results obtained by Setälä et al (1996) in their exhaustive and well-designed study help to clarify some of the on-going controversies regarding the prognostic significance of several factors in gastric carcinoma and emphasize the importance of data that may be 'easily obtained in connection with routine histological examination without any special or laborious techniques'. The study also represents a major breakthrough in the evaluation of the prognostic value of mitotic index in gastric carcinoma and clarifies the relationship between Laurén's classification and grading of tumours. We fully agree that diffuse-type carcinomas should not be graded into well-moderately and poorly differentiated carcinoma. This policy was unfortunately not followed in most of the reports on record in which isolated cell-diffuse carcinomas were considered, erroneously in our opinion, in the group of poorly differentiated carcinomas.

The main conclusions of the study are similar to those of our own studies: the extent of the neoplastic disease as measured according to the TNM criteria (pTNM) and vascular invasiveness are the most important prognostic factors in gastric carcinoma, despite the differences in the incidence of the latter from series to series (Ribeiro et al, 1988; Carneiro et al, 1995; Setälä et al, 1996). As in the study of Setälä et al (1996), lymphoid infiltration does not appear to be an independent prognostic predictor in multivariate analysis (Ribeiro et al, 1988; Carneiro et al, 1995). In our hands, patients with carcinomas showing moderate to abundant desmoplastic response do worse than patients with tumours lacking this histological feature (Ribeiro et al, 1988; Carneiro et al, 1995). The presence of moderate to abundant desmoplasia was significantly correlated with the size and shape of tumours, being particularly frequent in large tumours displaying an infiltrative or ulcero-infiltrative macroscopic appearance; despite these correlations, the evaluation of desmoplastic response was found to carry significant ($P < 0.02$) prognostic information in multivariate analysis in one of the aforementioned studies (Ribeiro et al, 1988).

In the study, Laurén's classification (Laurén, 1965) was significantly related to survival in the group of operated patients in a multivariate analysis. This finding does not entirely fit with the results obtained by our group (Ribeiro et al, 1988; Carneiro et al, 1995). In our experience, the data provided by Laurén's classification are not an independent prognostic predictor in patients submitted to potentially curative resection of gastric carcinoma (Ribeiro et al, 1988; Carneiro et al, 1995). On the other hand, the classification of gastric carcinoma we recently proposed – encompassing glandular carcinoma, isolated cell carcinoma, solid carcinoma and mixed carcinoma, and thus representing a sort of revised Laurén's classification – was found to carry an independent prognostic meaning (Carneiro et al, 1995). Although the reasons underlying these discrepancies are not clear, it is tempting to hypothesize that they can be ascribed, partly at least, to the different criteria used in the classification of the tumours. The comparison of the prevalences of the different types of tumours observed by Setälä et al (1996) (diffuse carcinoma – 44%; intestinal carcinoma – 44%; mixed carcinoma – 8%, unclassified carcinoma – 4%) and those observed by our group (Carneiro et al, 1995) (isolated cell carcinoma – 7%; glandular carcinoma – 42%; mixed carcinoma – 39%; solid carcinoma – 13%) highlights the difference in the prevalence of isolated cell–/diffuse carcinomas between the two series. In the belief that there are no major geographical differences in the incidence of the different types of gastric cancer, we feel tempted to advance the theory that the group of diffuse carcinomas in the series of Setälä et al (1996) encompasses both 'pure' diffuse carcinomas and tumours predominantly composed by diffuse type carcinoma, regardless of the existence of other minor histological component(s). We classify the latter cases as mixed carcinomas provided the minor component occupies more than 5% of the whole tumour. Using this criterion to classify the carcinomas of our series, we observed that the survival rate of patients with mixed tumours, regardless of the histological pattern of the predominant component, was significantly worse than those of patients with tumours composed by a single histological component (Carneiro et al, 1995). We proposed therefore that tumours with dual differentiation should be classified as a separate group (mixed carcinomas) carrying a guarded prognosis per se (Carneiro et al, 1995).

Taking into account the data of Iriyma et al (1993), Setälä et al (1996) proposed that the prognostic potential of Laurén's classification may be stage related, which could partly explain the disagreement between the results obtained from variable materials. We believe that this is a good working hypothesis that should be tested in a large series of tumours using not only the classification originally proposed by Laurén (1965), but also the revised version we have proposed (Cameiro et al, 1995).

*Fátima Carneiro*
*Manuel Moutinho Ribeiro*
*Manuel Sobrinho-Simões*
*IPATIMUP*
*Department of Pathology*
*Medical Faculty of Porto*
*4200 Porto*
*Portugal*

### REFERENCES

Carneiro F, Seixas M and Sobrinho-Simões M (1995) New elements for an updated classification of the carcinomas of the stomach. *Pathol Res Pract* **191**: 571–584

Iriyama K, Miki C, Ilunga K, Osawa T, Tsuchibashi T and Susuki H (1993) Prognostic significance of histological type in gastric carcinoma with invasion confined to the stomach wall. *Br J Surg* **80**: 890–892

Laurén P (1965) The two histological main types of gastric carcinoma: diffuse and so-called intestinal-type carcinoma. An attempt at a histoclinical classification. *Acta Pathol Microbiol Scand* **64**: 31–49

Ribeiro MM, Seixas M and Sobrinho-Simões M (1988) Prognosis in gastric carcinoma. The preeminence of staging and futility of histological classification. *Digest Dis Pathol* **1**: 51–68

Setälä LP, Kosma VM, Marin S, Lipponen PK, Eskelinen MJ, Syrjänen KJ and Alhava EM (1996) Prognostic factors in gastric cancer: the value of vascular invasion, mitotic rate and lymphoplasmacytic infiltration. *Br J Cancer* **74**: 766–772